# Correction to: A common gene drive language eases regulatory process and eco-evolutionary extensions

**DOI:** 10.1186/s12862-021-01909-3

**Published:** 2021-10-25

**Authors:** Prateek Verma, R. Guy Reeves, Chaitanya S. Gokhale

**Affiliations:** 1 Research Group for Theoretical Models of Eco‑evolutionary Dynamics, Department of Evolutionary Theory, Max Planck Institute for Evolutionary Biology, Plön, Germany; 2 Department of Evolutionary Genetics, Max Planck Institute for Evolutionary Biology, Plön, Germany

## Correction to: BMC Ecol Evol (2021) 21:156 https://doi.org/10.1186/s12862-021-01881-y

Following the publication of the original article [1], we were notified that Figs. 1, 3, 4 were incomplete.

- Originally published figures:
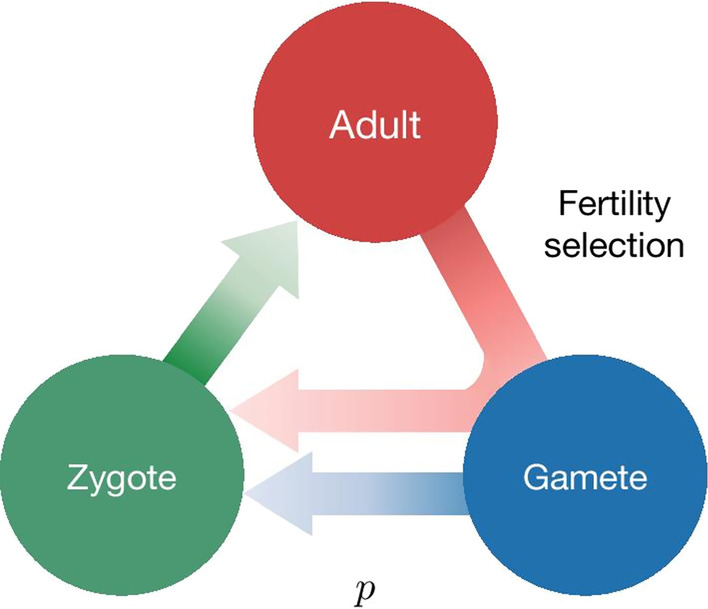

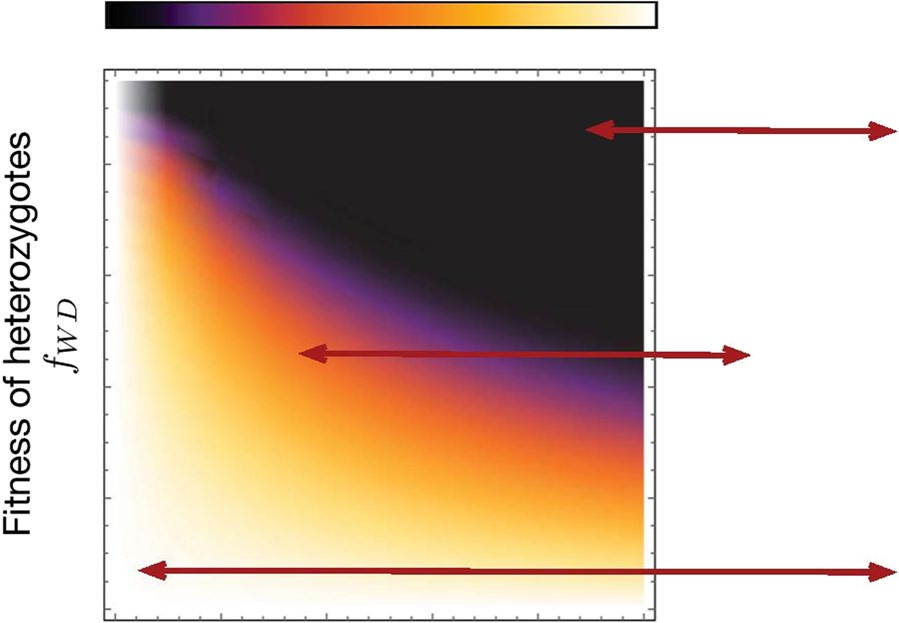

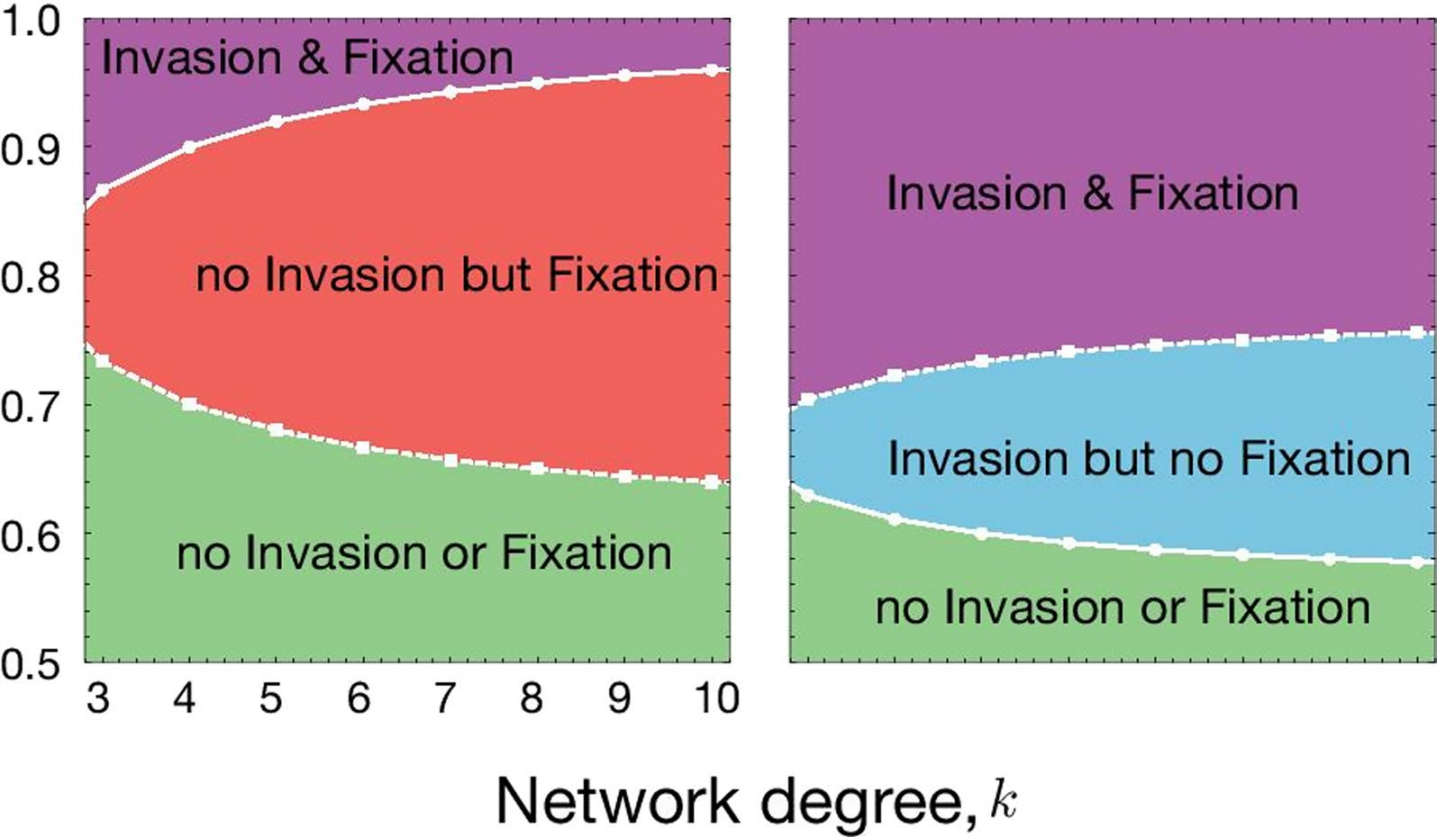

- Corrected figures (Figs. 1, 3, 4):

**Fig. 1:**
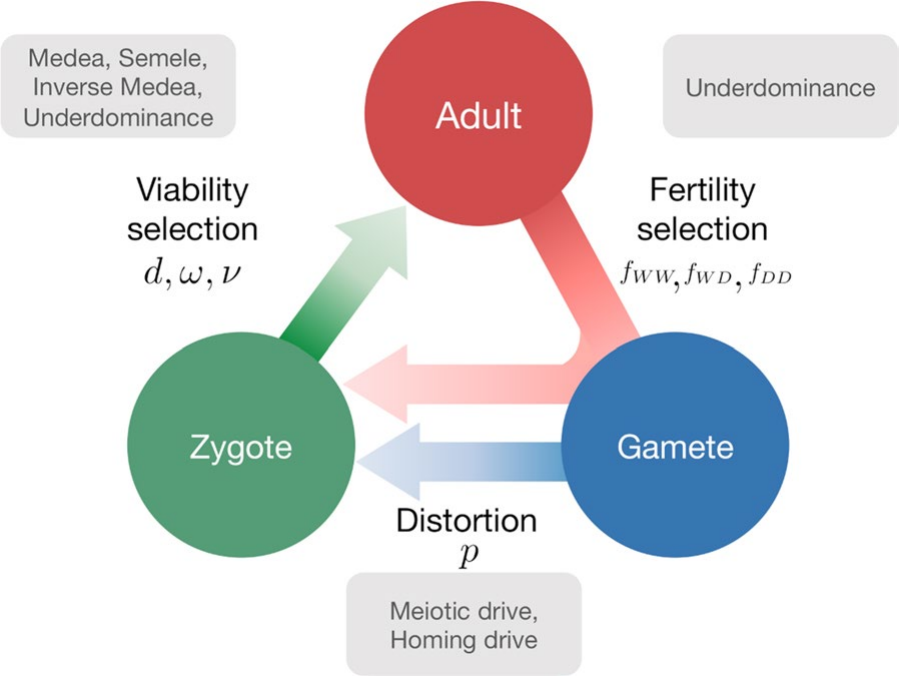
Lifecycle of an individual organism for a generic gene drive model. Assuming that individuals reproduce sexually and that the lifecycle has three stages, Adult, Gamete and Zygote. Adults produce gametes which combine to form zygotes. Zygotes grow up to become adults. Three factors can act during the life stages of an organism: distortion, viability selection and fertility selection (represented as arrows). Each can influence the probability of inheritance of a gene in the population and can be potentially manipulated to engineer gene drive constructs. Parameters, described in the text, are associated with each of the three arrows. Examples of named drive systems that can be generated are provided associated with the respective arrow

**Fig. 3:**
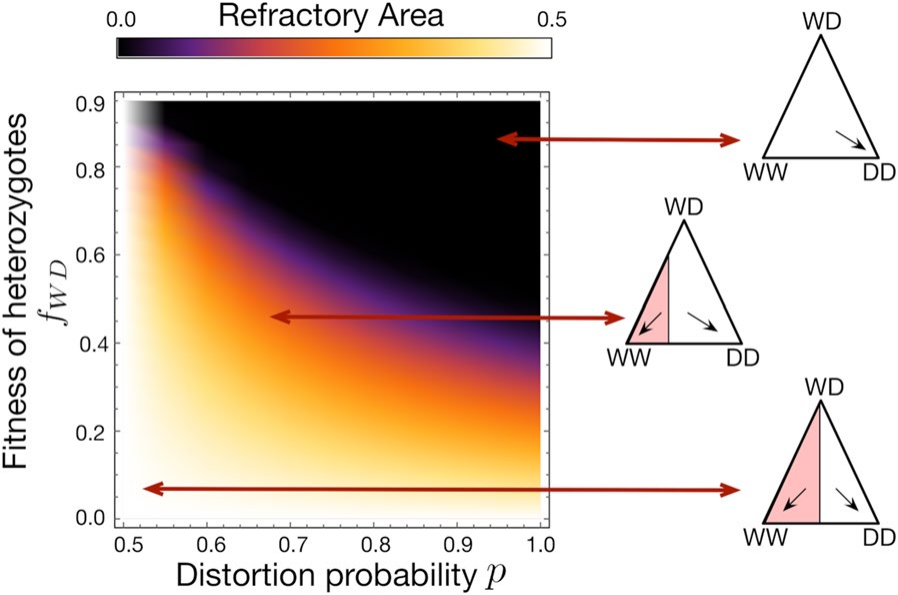
Heat-map showing the refractory zone with variation in distortion probability *p* and fertility fitness of heterozygotes *f*_*WD*_. Illustration of refractory zone for specific values of *p* and *f*_*WD*_ of the heat-map. Trajectories of a de Finetti diagram when 2*pf*_*WD*_ > *f*_*WW*_, drive individuals invade the wild population. Refractory zone is zero and is shown by black colour in the heatmap. *p* = 0.5 corresponds to’no distortion’ case. The values of other parameter is fixed to *f*_*WW*_ = 1, *f*_*DD*_ = 1

**Fig. 4:**
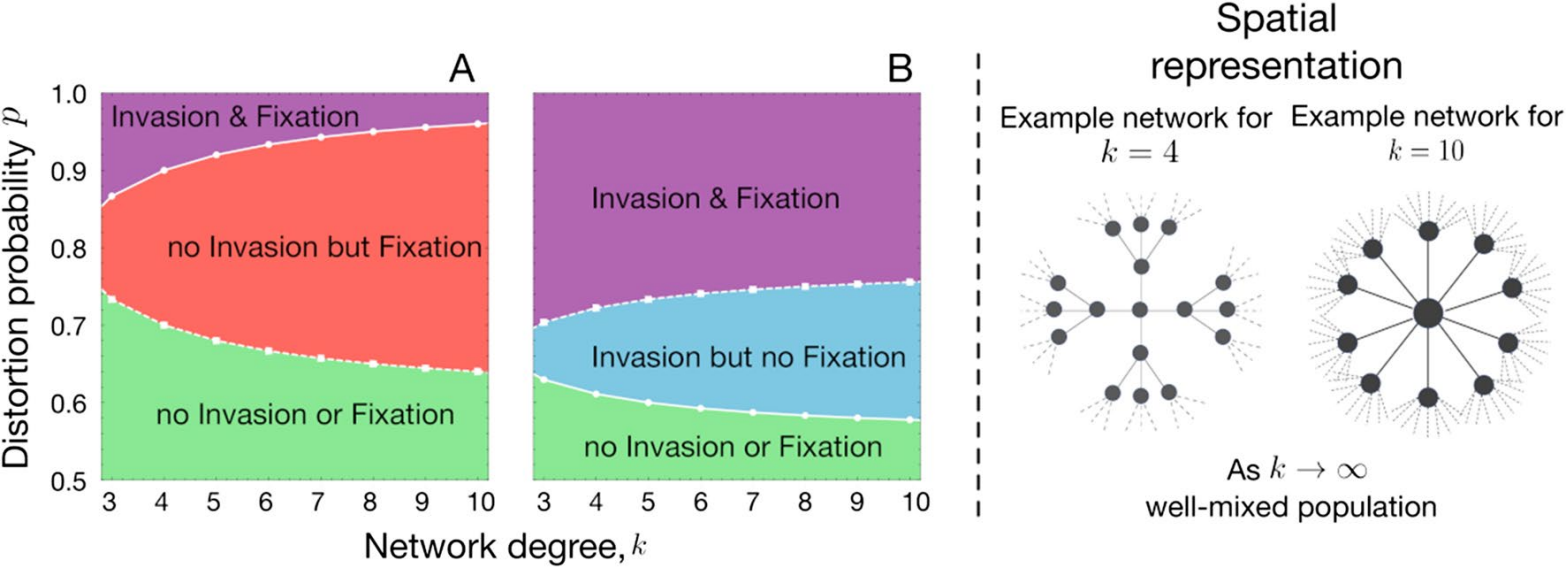
Spatial structure affects the condition for the invasion from rare and fixation of the driven gene. **A** Variation in invasion (full line with circles) and fixation (dashed line with squares) conditions with respect to network degree (*k*) and distortion parameter (*p*) for *f*_*WD*_ = 0.5 and **B** *f*_*WD*_ = 0.9. The values of other parameters are fixed to *f*_*WW*_ = 1, *f*_*DD*_ = 0.4. Population dynamics changes when the population becomes more structured on the Bethe lattice parameterized by *k*. Lower *k* means more structured population and higher *k* represents less structure (closer to well-mixed case). The change in population dynamics properties can be seen by the change in invasion/fixation condition and combinations of them, such as no invasion from rare but fixation, if sufficient drive individuals are released/migrate

The original article has been corrected.
